# Emodin Induces Apoptotic Death in Murine Myelomonocytic Leukemia WEHI-3 Cells *In Vitro* and Enhances Phagocytosis in Leukemia Mice *In Vivo*

**DOI:** 10.1155/2011/523596

**Published:** 2011-05-10

**Authors:** Yuan-Chang Chang, Tung-Yuan Lai, Chun-Shu Yu, Hung-Yi Chen, Jai-Sing Yang, Fu-Shin Chueh, Chi-Cheng Lu, Jo-Hua Chiang, Wen-Wen Huang, Chia-Yu Ma, Jing-Gung Chung

**Affiliations:** ^1^School of Chinese Medicine, China Medical University, Taichung 404, Taiwan; ^2^School of Post-Baccalaureate Chinese Medicine, China Medical University, Taichung 404, Taiwan; ^3^Department of Chinese Medicine and Chinese Internal Medicine, China Medical University Hospital, Taichung 404, Taiwan; ^4^School of Pharmacy, China Medical University, Taichung 404, Taiwan; ^5^Department of Pharmacology, China Medical University, Taichung 404, Taiwan; ^6^Department of Health and Nutrition Biotechnology, Asia University, Taichung 412, Taiwan; ^7^Department of Life Sciences, National Chung Hsing University, Taichung 402, Taiwan; ^8^Department of Biological Science and Technology, China Medical University, Taichung 404, Taiwan; ^9^Department of Food and Beverage Management, Technology and Science Institute of Northern Taiwan, Taipei 112, Taiwan; ^10^Department of Biotechnology, Asia University, Taichung 412, Taiwan

## Abstract

Emodin is one of major compounds in rhubarb (*Rheum palmatum* L.), a plant used as herbal medicine in Chinese population. Although many reports have shown that emodin exhibits anticancer activity in many tumor cell types, there is no available information addressing emodin-affected apoptotic responses in the murine leukemia cell line (WEHI-3) and modulation of the immune response in leukemia mice. We investigated that emodin induced cytotoxic effects *in vitro* and affected WEHI-3 cells *in vivo*. This study showed that emodin decreased viability and induced DNA fragmentation in WEHI-3 cells. Cells after exposure to emodin for 24 h have shown chromatin condensation and DNA damage. Emodin stimulated the productions of ROS and Ca^2+^ and reduced the level of ΔΨ_*m*_ by flow cytometry. Our results from Western blotting suggest that emodin triggered apoptosis of WEHI-3 cells through the endoplasmic reticulum (ER) stress, caspase cascade-dependent and -independent mitochondrial pathways. In *in vivo* study, emodin enhanced the levels of B cells and monocytes, and it also reduced the weights of liver and spleen compared with leukemia mice. Emodin promoted phagocytic activity by *monocytes* and *macrophages* in comparison to the leukemia mice group. In conclusions, emodin induced apoptotic death in murine leukemia WEHI-3 cells and enhanced phagocytosis in the leukemia animal model.

## 1. Introduction

Emodin (1,3,8-trihydroxy-6-methylanthraquinone) is one of the major compound in the root of rhubarb (*Rheum palmatum* L.) [1, 2] and possesses immunosuppressive, anticancer, antiinflammatory, antiatherosclerotic, vasorelaxant, and vasorelaxant effects [3–5]. Numerous reports have shown that emodin has antiproliferative effects on many kinds of cancer cell lines such as HER-2/neu-overexpressing breast cancer [6, 7], leukemia [8, 9], hepatoma [10], and lung [11] cancer cells. Also, emodin-triggered apoptosis is mediated through the caspase- and mitochondria-dependent pathways in proximal tubular epithelial HK-2 cells [12]. Emodin enhanced gefitinib-induced cytotoxicity *via *Rad51 downregulation and ERK1/2 inactivation in human breast cancer BCap-37 cells [13]. Therefore, emodin has now been proposed as a potential agent in the management of tumors [14]. In our laboratory, we also found that emodin induced apoptosis in human tongue squamous cancer SCC-4 cells through reactive oxygen species (ROS) and mitochondria-dependent pathways [15], but it has cytotoxic and protective effects in rat C6 glioma cells, and Mdr1a and nuclear factor kappaB play important roles in cell survival [16].

Leukemia is one of the major causes of deaths in the human population. In Taiwan, about 4.0 per 100,000 people die each year of leukemia and it is the 11th most common malignancy based on the report of the Department of Health, Taiwan in 2009 [17]. In the clinical therapy, the major strategies for patients with leukemia including bone marrow transplant, radiotherapy, and chemotherapy [18, 19]. However, the cure rate and side effects are still unsatisfied, and to find new agent for leukemia patients is urgent. Numerous clinical drugs for cancer patients are obtained from natural products. Although various studies of biological activities on emodin have been carried out, regarding the molecular mechanisms of emodin-acted the cell death *in vitro* and promotion of immune responses in animal model *in vivo* are still undefined. Therefore, we investigated the effects of emodin on growth inhibition and apoptotic cell death of murine WEHI-3 leukemia cells *in vitro*, and modulation of immune responses in leukemia mice model* in vivo*.

## 2. Material and Methods

### 2.1. Reagents and Antibodies

Dimethyl sulfoxide (DMSO), *N*-acetylcysteine (NAC), propidium iodide (PI), Triton X-100, and RNase A were obtained from Sigma-Aldrich Corp. (St. Louis, MO, USA). The pan-caspase inhibitor (Z-VAD-FMK), caspase-3 inhibitor (Z-DEVD-FMK), and caspase-9 inhibitor (Z-LEHD-FMK) were purchased from R&D Systems (Minneapolis, Menn, USA). All primary antibodies were obtained from Santa Cruz Biotechnology, Inc. (Santa Cruz, Calif, USA). The peroxidase-conjugated secondary antibodies were purchased from Santa Cruz Biotechnology, Inc. Enhanced chemiluminescence (ECL) kit and Western blotting reagents were purchased from Pierce Chemical Co. (Rockford, Ill, USA).

### 2.2. In Vitro Studies

#### 2.2.1. Murine Leukemia Cell Line

WEHI-3 murine myelomonocytic leukemia cell line was purchased from the Food Industry Research and Development Institute (Hsinchu, Taiwan). Cells were seeded in 75-cm^2^ cell culture flasks and maintained in RPMI 1640 medium supplemented with 10% fetal bovine serum (FBS), 2 mM L-glutamine 100 U/mL penicillin, and 100 *μ*g/mL streptomycin at 37°C under a humidified 5% CO_2_ atmosphere.

#### 2.2.2. Cell Growth Inhibition Assay

The viable WEHI-3 cells were assessed by the MTT assay. About 1 × 10^4^ cells/100 *μ*L per well plated in 96-well plates were treated with emodin at 0, 25, 50, 100, and 150 *μ*M for 24 and 48 h. MTT solution (Sigma-Aldrich Corp., 5 mg/mL) was prepared and a volume of 10 *μ*L was individually added to each well for 4-h incubation [20]. MTT is reduced to form purple formazan product by the mitochondrial dehydrogenases of viable cells. The MTT-purple formazan productions were dissolved in DMSO and then were measured by absorbance at 570 nm in an ELISA plate reader as described previously [20].

#### 2.2.3. DNA Laddering Fragmentation

Approximately 2 × 10^5^ cells/well of WEHI-3 cells were grown in 12-well plates and treated with 0, 50, 100, and 150 *μ*M of emodin for 24 h. DNA was isolated from emodin-treated and untreated cells and then were measured in 1.5% agarose gel electrophoresis, followed to photograph after being stained with ethidium bromide (Sigma-Aldrich Corp.) under UV illumination as described previously [21].

#### 2.2.4. DAPI Staining for Apoptosis

Cells (2 × 10^5^ cells/well) were seeded in 12-well plates and emodin was individually added to the cells at final concentrations at 0, 50, and 100 *μ*M for 24 h. Cells were harvested, washed with PBS, and fixed with 4% paraformaldehyde. The fixed cells were then washed with PBS and stained with 4,6-diamidino-2-phenylindole (DAPI, 1 *μ*g/mL; Invitrogen) for 30 min in the dark. Cells were then examined and determined under a fluorescent microscope, photographed and apoptotic cells identified [22, 23].

#### 2.2.5. Comet Assay for DNA Damage

The Comet assay was followed the procedures of Wang et al. with some modifications [24]. Cells (2 × 10^5^ cells/well) in 12-well plates were incubated with 0, 25, 50, and 100 *μ*M of emodin for 24 h. Cells were harvested for the examination of DNA damage using the Comet assay. Comets of cells on slides acquired DNA damage by using the CometScore Freeware analysis (TriTek Corporation, Sumerduck, VA, USA). Comet tail length was calculated, quantified, and expressed in mean ± S.D. as previously described [23].

#### 2.2.6. Detection of Reactive Oxygen Species (ROS), Ca^2+^ Production Levels and Mitochondrial Membrane Potential (ΔΨ_*m*_)

Cells (2 × 10^5^ cells/mL) in RPMI 1640 medium were treated with 100 *μ*M of emodin for 0, 1, 3, 6, 12, or 24 h. The cells were harvested, washed twice with PBS, and then resuspended in 500 *μ*L of 2,7-dichlorodihydrofluorescein diacetate (10 *μ*M) (DCFH-DA) for determining the changes in ROS, in 500 *μ*L of Fluo-3/AM (2.5 *μ*g/mL) for staining of Ca^2+^ and in 500 *μ*L of DiOC_6_ (500 nmol/L) for determining ΔΨ_*m*_. Cells then were individually incubated at 37°C for 30 min and were analyzed by flow cytometry (Becton Dickinson FACSCalibur, San Jose, Calif, USA) and BD CellQuest Pro software [21, 23, 25].

#### 2.2.7. Determinations for Caspase-3, -8 and -9 Activity

Cells (1 × 10^6^ cells/mL) in RPMI 1640 medium in 10-cm dishes were exposed to 100 *μ*M of emodin for 24 h or pretreated with a pan-caspase inhibitor (10 *μ*M, Z-VAD-FMK) for 3 h. The cells were harvested and lysed in lysis buffer (50 mM Tris-HCl (pH 7.4), 1 mM EDTA, 10 mM EGTA, 10 mM digitonin and 2 mM DTT). The cell lysates (50 *μ*g proteins) were incubated with caspase-3, -9, and -8 specific substrates (Ac-DEVD-pNA, Ac-LEHD-pNA, and Ac-IETD-pNA) (R&D systems Inc., Minneapolis, MN, USA) for 1 h at 37°C. The caspases activities were determined by measuring the release of pNA at OD_405_ as previously described [26, 27]. Cells were also pretreated with the caspase-9 inhibitor (10 *μ*M, Z-IETD-FMK), caspase-3 inhibitor (10 *μ*M, Z-DEVD-FMK) (R&D Systems) and NAC, and then were treated with 100 *μ*M emodin for 24-h exposure. Cells were harvest for determination of viability as previously described [28, 29].

#### 2.2.8. Western Blotting for Examining the Apoptosis-Associated Protein Levels

Cells (2 × 10^5^ cells/mL) in RPMI 1640 medium were treated with 100 *μ*M of emodin for 0, 6, 12, and 24 h, isolated cells from each treatment were lysed and the protein levels were determined as described previously [15, 25] for determining apoptosis-associated proteins levels such as caspase-3, -7 and -9, PARP, cytochrome *c*, Apaf-1, AIF, Endo G, GADD153, GRP78, ATF-6*α*, Bcl-2, Bcl-xL, Bax and Bad. All samples were separated by sodium dodecyl sulfate polyacrylamide (SDS-PAGE) gel electrophoresis as described previously [15, 25]. Quantification of band density was determined using NIH Image J software.

### 2.3. In Vivo Studies

#### 2.3.1. BALB/c Mice

Fifty male BALB/c mice, each was approximately 22–28 g in body weight at 8 weeks of age were purchased from the Laboratory Animal Center, College of Medicine, National Taiwan University (Taipei, Taiwan). These animals were maintained at 25°C on a 12-h light/dark cycle in the animal center of the China Medical University followed the animal guideline as previously described [30, 31].

#### 2.3.2. Emodin Treatment

Fifty mice were randomly divided into 5 groups (10 animals per group). Group I was control (normal animal) and Group II was only treated olive oil. Group III was intraperitoneally (i.p.) injected with WEHI-3 cells (1 × 10^5^ cells) and the olive oil treatment only as negative control. Group IV was i.p. injected with WEHI-3 cells (1 × 10^5^ cells) and orally treated with emodin (5 mg/kg) in olive oil. Group V was i.p. injected with WEHI-3 cells (1 × 10^5^ cells) and oral treatment with emodin (10 mg/kg) in olive oil [8, 32]. Emodin (5 and 10 mg/kg) was administered by oral gauge in 100 *μ*L of olive oil. Animals were inoculated with WEHI-3 cells for 2 weeks of incubation, and subsequently leukemia mice were administrated daily for 2 weeks before being weighed and sacrificed. 

#### 2.3.3. Blood Collection and Immunofluorescence Staining

After emodin treatment for 2 weeks, one mL of blood was collected from each mouse of examined groups. The individual samples were added 1 × Pharm Lyse lysing buffer (BD Biosciences, San Jose, CA, USA) for lysing of the red blood cells and then centrifuged at 1000 × g at 4°C for 15 min. Isolated white blood cells were stained with anti-CD3-fluorescein isothiocyanate (FITC), -CD11b-FITC, -CD19-phycoerythrin (PE), and -Mac-3-PE antibodies (BD Pharmingen, San Diego, CA, USA) for measuring the cell surface markers of T cell (CD3), B cell (CD19), monocytes, and macrophages (CD11b and Mac-3, resp.) and then were determined the cell marker levels by flow cytometry as previously described [30, 33].

#### 2.3.4. Phagocytic Activity of Monocytes and Macrophages

Leukocytes from the mice were collected for determining the phagocytosis by using a PHAGOTEST kit (Glycotope Biotechnology GmbH/Orpegen Pharma, Heidelberg, Germany) as previously described [30, 31]. Cells were isolated from peripheral blood mononuclear cells (PBMC), and peritoneal cavity of control and emodin-treated animals. Isolated cells were individually incubated with opsonised FITC-labeled *E. coli* (20 *μ*L) for 1 h at 37°C according to the manufacturer's instruction. After incubation, an ice-cold quenching solution (100 *μ*L) was added to stop the reaction then and DNA content of the monocytes/macrophages for cell cycle analysis. Cells were prepared then were analyzed by flow cytometery. Fluorescence data were collected on 10,000 cells by flow cytometry and analyzed using the BD CELLQUEST Pro software [30, 31].

### 2.4. Statistical Analyses

Data were expressed as mean ± SD and differences between control and emodin-treated groups were analyzed by one-way ANOVA followed by Bonferroni's test for multiple comparisons or Dunnett's test. *P < .05 and ***P < .001 were considered significant.

## 3. Results

### 3.1. Emodin Induced Cytotoxic Effects and DNA Fragmentation in WEHI-3 Cells

**Figure 1:**
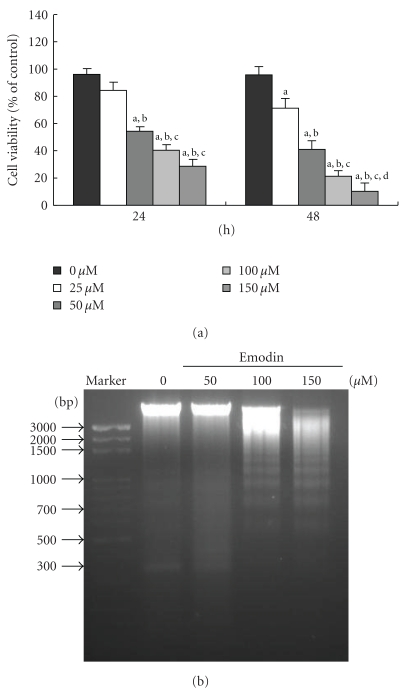
Emodin affected the percentage of viable cells and DNA fragmentation in WEHI-3 cells. Cells (1 × 10^4^ cells/well; 96-well plates) were plated in RPMI 1640 medium + 10% fetal bovine serum (FBS) with 0, 25, 50, 100, and 150 *μ*M of emodin for 24 and 48 h. The cells were collected by centrifugation and the viable cells were determined by using the MTT assay (a). Cells were treated with 0, 50, and 100 *μ*M of emodin for 24 h, and then DNA was isolated for DNA gel electrophoresis (b) as described in Section 2. Columns, mean of three determinations; bars, SD. a, *P* < .05 shows significantly different when compared with DMSO-treated control; b, c, and d, *P* < .05 indicates significantly different compared with 25, 50, and 100 *μ*M emodin-treated groups, respectively (one-way ANOVA followed by Bonferroni's test for multiple comparisons).

The doses of emodin (50 to 150 *μ*M) are required to induce cell death in WEHI-3 cells for 24-h treatment and the percentage of viable cells decrease was 41–63% (Figure 1(a)). However, emodin (25–150 *μ*M) for 48-h treatment decrease viable cells and the results show 19–82% (Figure 1(a)). Herein, emodin in the concentration as low as 25 *μ*M initiated cell death of WEHI-3 cells and the IC_50_ is 100 *μ*M (Figure 1(a)). DNA gel electrophoresis assay indicated that 100–150 *μ*M of emodin-induced DNA fragmentation in WEHI-3 cells after exposure for 24 h (Figure 1(b)). 

### 3.2. Emodin Induced Apoptosis and DNA Damage in WEHI-3 Cells

**Figure 2:**
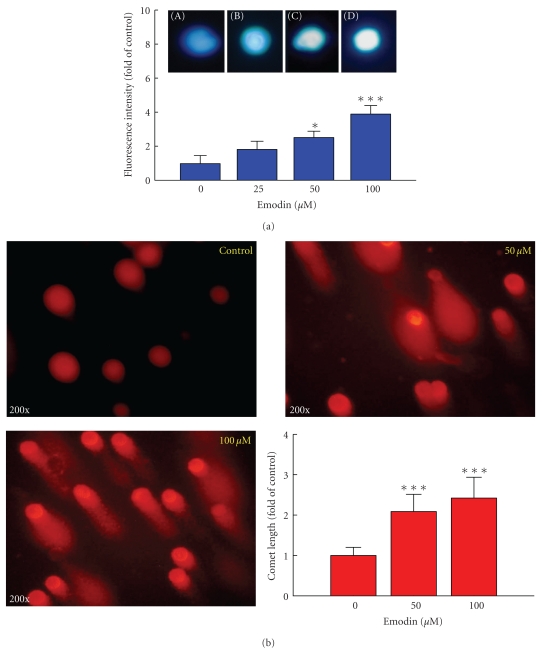
Effects of emodin on apoptosis and DNA damage in WEHI-3 cells by using DAPI staining and Comet assay. Cells (2 × 10^5^ cells/well) in 12-well plate were incubated with 0, 25, 50, and 100 *μ*M emodin for 24 h and apoptosis was determined using DAPI staining (a). Data represent mean ± SD of at least three experiments. Cells were treated with 0, 50, and 100 *μ*M emodin for 24 h and then were harvested for the examination of DNA damage using the Comet assay (b) as described in Section 2. Comet tail length was calculated, quantified and expressed in mean ± S.D for at least three replicates. *Significantly different compared with DMSO-treated control, *P* < .05, and ***significantly different from the control sample at *P* < .001.

To investigate the mechanism of emodin-induced leukemic cell death, the effect of emodin on DNA damage was evaluated. The effects of emodin on DNA integrity were further analyzed using the DAPI staining and Comet assay. Emodin-induced chromatin condensation (an apoptotic characteristic) in a dose-dependent response (Figure 2(a)). Also, emodin triggered DNA damage after 24-h treatment and quantification of the number of leukemic WEHI-3 cells displaying a Comet tail strongly increased after emodin treatment (Figure 2(b)).

### 3.3. Emodin Altered the Levels of ROS, Ca^2+^ and ΔΨ_*m*_ in WEHI-3 Cells

**Figure 3:**
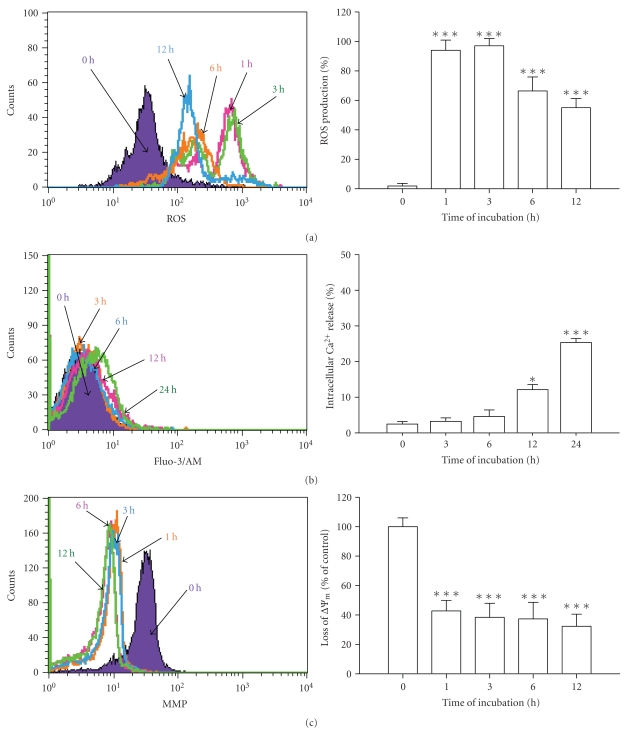
Emodin affected the levels of ROS, Ca^2+^ and ΔΨ_*m*_ in WEHI-3 cells. Cells were cultured in 100 *μ*M emodin for 0, 1, 3, 6, 12 or 24 h. Cells were harvested and resuspended in DCFH-DA for determining the changes in ROS (a), in Fluo-3/AM for staining of Ca^2+^ (b) and in DiOC_6_ for determining ΔΨ_*m*_ (c) as described in Section 2. *P < .05 and ***P < .001 were considered significant when compared with DMSO-treated control.

Cells treated with 100 *μ*M emodin were determined by flow cytometric assays and results are shown in Figures 3(a), 3(b), and 3(c), which indicated that emodin promoted the levels of ROS (Figure 3(a)) and Ca^2+^(Figure 3(b)), but decreased the level of ΔΨ_*m*_ (Figure 3(c)). To investigate emodin-induced cell death is through the disruption of mitochondrial respiratory chain, leading to ROS accumulation and cellular damage. An increase in intracellular fluorescence after DCFH-DA loading showed the reversal of ROS accumulation (Figure 3(a)). In order to elucidate the mechanism of emodin-induced mitochondria-dependent apoptotic cell death, the effects of emodin on the levels of intracellular Ca^2+^ and ΔΨ_*m*_ were analyzed. These data indicated that emodin-induced apoptosis is possibly mediated through alteration of mitochondrial permeability transition in WEHI-3 cells *in vitro*.

### 3.4. Emodin Affected Activities of Caspase-3 and -9 of WEHI-3 Cells

**Figure 4:**
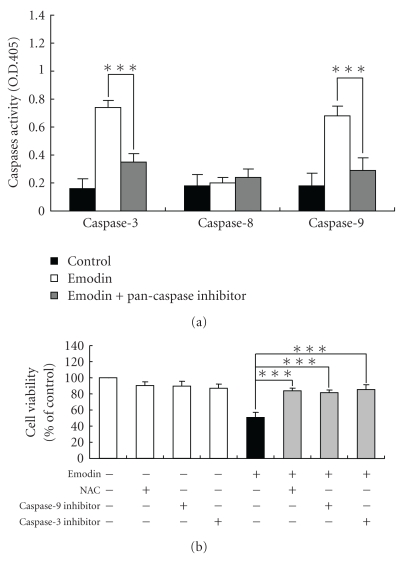
Emodin stimulated the activities of caspase-3 and -9 of WEHI-3 cells. Cells were seeded in RPMI 1640 medium + 10% FBS with pretreatment with NAC, inhibitors of caspase-9 and -3 or a pan-caspase inhibitor, and then were exposed to 100 *μ*M of emodin for 24 h. Cells were determined the caspase-3, -8, and -9 activity (a) and percentage of viable cells (b) as described in Section 2. Each experiment was done with triple sets, and columns, mean of three determinations; bars, SD. *P < .05 and ***P < .001 were considered significant different as compared to the DMSO-treated control group.

In order to evaluate the roles of caspase-mediated pathways in emodin-induced apoptotic death of WEHI-3 cells, cells were pretreated with a pan-caspase inhibitor (Z-VAD-FMK), and then exposed to emodin. We found that emodin promoted the activities of caspase-3 and -9, but it did not affect that of caspase-8 (Figure 4(a)). Pretreatment with Z-VAD-FMK could decrease emodin-stimulated the activities of caspase-3 and -9 in WEHI-3 cells (Figure 4(a)). Furthermore, cells were individually preincubated with an antioxidant scavenger (NAC), caspase-9 inhibitor (Z-IETD-FMK), and caspase-3 inhibitor (Z-DEVD-FMK), which led to increase emodin-reduced the percentage of viable WEHI-3 cells compared to the emodin-treated cells only (Figure 4(b)). These data suggest that emodin triggered apoptosis of WEHI-3 cells through the ROS and caspase-dependent pathways. 

### 3.5. Emodin Altered the Apoptosis-Associated Protein Levels in WEHI-3 Cells

**Figure 5:**
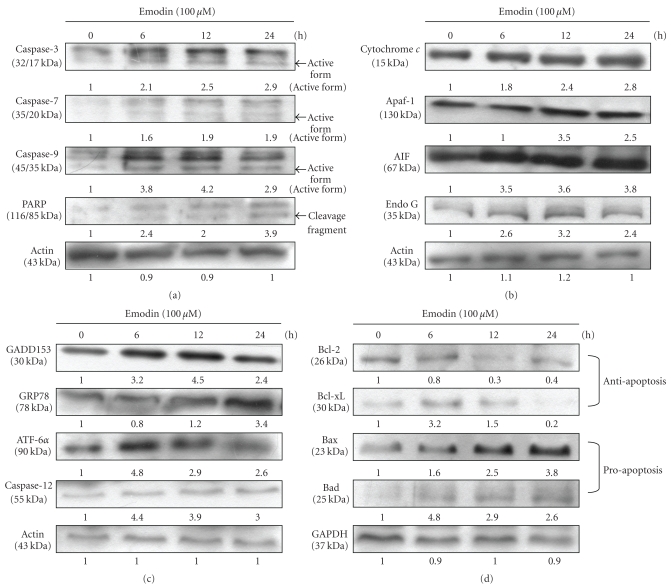
Emodin affected the apoptosis-associated protein levels in WEHI-3 cells. Cells were treated with emodin at 100 *μ*M for 0, 6, 12, and 24 h, and then the total proteins were prepared and determined as described in Section 2. The levels of associated proteins expressions ((a): caspase-3, -7, and -9 and PARP; (b): cytochrome *c*, Apaf-1, AIF and Endo G; (c): GADD153, GRP78, ATF-6*α* and caspase-12; (d): Bcl-2, Bcl-xL, Bax, and Bad) were estimated by Western blotting as described in Materials and Methods.

**Figure 6:**
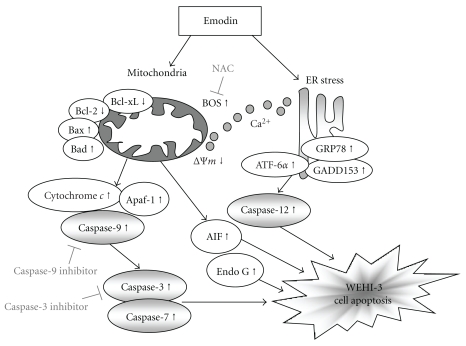
The proposed mechanisms of emodin-induced apoptosis in WEHI-3 cells. The flow chart shows that emodin induced apoptosis through the ER stress, mitochondria-, and caspase-3-dependent signaling pathways in murine leukemia WEHI-3 cells *in vitro*.

Cells were exposure to 100 *μ*M of emodin for 0, 6, 12, and 24 h and then the total protein were prepared and determined by Western blotting analysis. These results are presented in Figure 5 ((a): caspase-3, -7 and -9, PARP; (b): cytochrome *c*, Apaf-1, AIF, Endo G; (c): GADD153, GRP78, ATF-6*α*, caspase-12; (d): Bcl-2, Bcl-xL, Bax, and Bad), which indicated that the levels of caspase-3, -7 and -9, PARP (Figure 5(a)), cytochrome c, Apaf-1, AIF, Endo G (Figure 5(b)), GADD153, GRP78, ATF-6*α*, caspase-12 (Figure 5(c)), Bax, and Bad (Figure 5(d)) were increased, while the levels of Bcl-2 and Bcl-xL (Figure 5(d)) were decreased and these effects may lead to cell apoptosis. Overall, the proposed possible signal pathways for emodin-induced apoptosis are shown in Figure 6.

### 3.6. Emodin Affected the Weight of BALB/c Mice after Injection with WEHI-3 Cells and/or Exposure to Emodin for 2 Weeks

**Figure 7:**
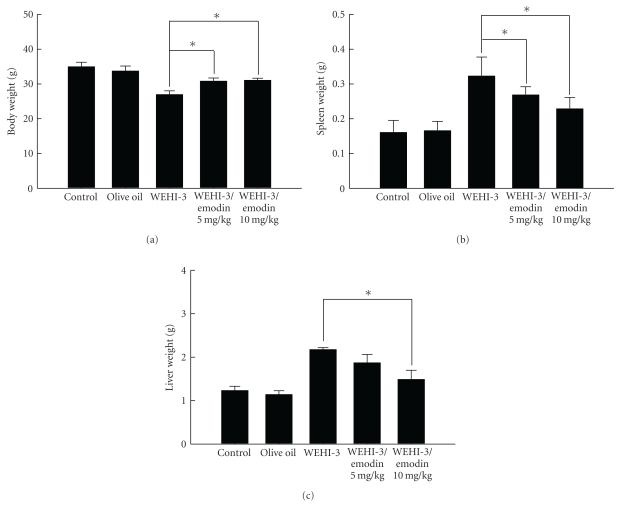
Emodin affected the weights of the leukemia mice which were treated with without or with emodin 2 weeks. BALB/c mice were intraperitoneally injected with WEHI-3 cells (1 × 10^5^ cells/100 *μ*L) in PBS for 2 weeks and/or treated with emodin once daily by oral administration for 14 days. Blood was collected and animals were sacrificed for examinations of weights of body (a) spleen (b) and liver (c) tissues, and then were individually weighed. Each point is the mean ± SD and similar results were observed in at least three independent experiments (*n* = 10) followed by one-way ANOVA followed by Dunnett's test.

The BALB/c mice after injection with WEHI-3 cells were treated with or without emodin (5 and 10 mg/kg). Results indicate that emodin increased the body weight of mice (Figure 7(a)) but decreased the weights of spleen (Figure 7(b)) and liver (Figure 7(c)) when compared with the leukemia mice group.

### 3.7. Emodin Affected the Cell Markers of White Blood Cells from Leukemia BALB/c Mice

**Figure 8:**
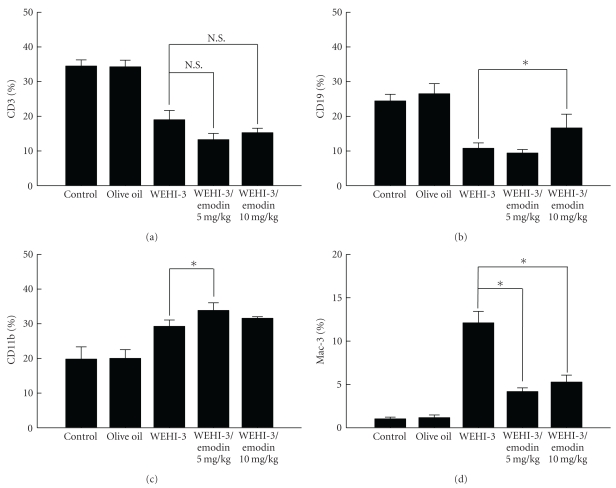
Emodin affected the cell markers of white blood cells from leukemia BALB/c mice. The leukemia mice were orally treated without and with emodin (5 and 10 mg/kg) in olive oil for 14 days. Blood was collected from individual animals and was analyzed for surface cell markers by flow cytometry as described in Section 2. Each point is the mean ± SD and similar results were observed in at least three independent samples (*n* = 10) followed by one-way ANOVA followed by Dunnett's test. N.S. = Not Significant (*P* > .05).

The leukemia mice were orally treated without (control) or with emodin (5 and 10 mg/kg) for 14 days. Blood was collected individually from animals of each group and was analyzed for cell markers by flow cytometry. The results are shown in Figures 8(a), 8(b), 8(c), and 8(d). Emodin did not affect the level of CD3 surface marker (Figure 8(a)); however, emodin increased the levels of CD19 (Figure 8(b)) and CD11b (Figure 8(c)) in emodin-treated groups and it also decreased the level of Mac-3 marker after emodin at 5 and 10 mg/kg treatment (Figure 8(d)) in comparison to leukemia mice group.

### 3.8. Emodin Affected on Phagocytosis by Monocytes and Macrophages from BALB/c Mice

**Figure 9:**
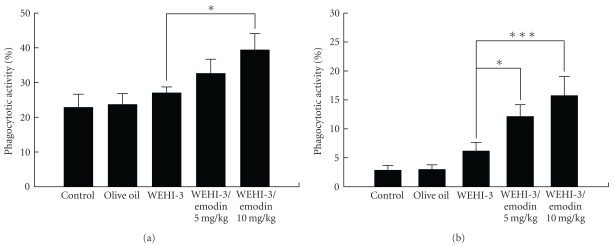
Emodin affected on phagocytosis by monocytes and macrophages from leukemia BALB/c mice. Mice were injected with WEHI-3 cells (1 × 10^5^ cells/100 *μ*L) in PBS for 2 weeks and treated without or with emodin for 2 weeks. Leukocytes were collected from PBMC (a) and peritoneal cavity (b) from animals and were analyzed for phagocytosis by flow cytometry as described in Section 2. Each point is the mean ± SD and similar results were observed in at least three independent samples (*n* = 10), and *P < .05 and ***P < .001 (*n* = 10) were shown significant followed by one-way ANOVA followed by Dunnett's test.

Animals were injected with WEHI-3 cells for 2 weeks and then treated without or with emodin for 2 weeks. Leukocytes were collected from PBMC and the peritoneal cavity and were analyzed for phagocytosis of monocytes and macrophages by flow cytometry. Results shown in Figures 9(a) and 9(b) indicate that emodin promoted the phagocytosis from PBMC and peritoneal cavity of leukemia mice.

## 4. Discussion

The major differences between leukemic cells and tumors are that leukemic cells capable of circulating and having access to various organs through interaction with activated vascular cells [34]. However, the subsets of leukemic cells may also adhere to vascular cells for establishing perivascular infiltrates then may be endowed with a unique mechanism of resistance to chemotherapy. It is well documented that circulating and vascular-adherent leukemic cells require cytoskeletal stability for maintaining mitochondrial and cellular function to avoid cell death [35]. In this study, we show that various doses of emodin selectively induced apoptosis of murine WEHI-3 leukemic cells by caspase-dependent response as well as ROS-mediated signal pathways for leading to cell death. Moreover, we also found that emodin-triggered apoptotic death is involved in the unfolded protein response (endoplasmic reticulum stress) in WEHI-3 cells. The molecular mechanisms of emodin-caused cell death are complex and most likely mediated through recruitment of caspase-dependent and -independent pathways.

In leukemia cells, various concentrations of emodin have been shown to result in caspase activations and apoptotic cell death [9, 36]. Emodin also induced G2/M phase arrest and subsequent cell death in leukemia cells [9]. However, our data showed that emodin impaired leukemic cell survival by caspases activation and in part through ROS production, Ca^2+^ release and the disruption of ΔΨ_*m*_ and releases of some proapoptotic factors including cytochrome *c*, AIF and Endo G. Mitochondrial dysfunction might result in the release of cytochrome *c*, AIF and Endo G in emodin-treated WEHI-3 cells as can be seen in Figure 5.

Mitochondrial membrane potential disruption and G2/M arrest have previously been described in leukemia cells upon treatment with emodin or aloe-emodin and rhein from rhubarb (Da-huang) [8, 9, 37, 38]. In the present study, our data suggest that emodin induced leukemic cell death by an apoptotic pathway that is distinct from conventional other compounds agents. We also examined whether emodin-induced mitochondrial damage promoted the release of cytochrmoe *c*, AIF and Endo G (Figure 5(b)). AIF and Endo G that are ubiquitously expressed flavoproteins, which might play a critical role in caspase-independent apoptosis [39, 40]. It was reported that AIF is similar to cytochrome *c* normally localized to the mitochondrial intermembrane space and released in response to apoptotic stimuli [41]. Our results also showed that emodin promoted the level of Bax, and decreased the level of Bcl-2 (Figure 5(c)), and it is well documented that the ratio of Bax/Bcl-2 involved the dysfunction of mitochondria, resulting in cell apoptosis [42]. Bcl-2, an upstream effector molecule in the apoptotic pathway, has been recognized to be a potent suppressor of apoptosis [43], and most cancers generally overexpress Bcl-2 [44, 45]. In the present study, we observed that emodin significantly downregulated Bcl-2 protein and up-regulated the level of Bax protein in WEHI-3 cells (Figure 5(d)), suggesting that the involvement of an intrinsic apoptotic pathway is mediated in emodin-induced apoptosis in WEHI-3 cells.

Our results also showed that emodin promoted ROS and Ca^2+^ production in WEHI-3 cells (Figure 3). We also used DAPI staining and Comet assay to show that emodin induced DNA damage in WEHI-3 cells (Figure 4). As the increased ROS accumulation and DNA damage have been identified as mediators or initiators of apoptosis in certain condition [46]. These data indicated that emodin-induced cell death in WEHI-3 cells is mediated *via* loss of ΔΨ_*m*_, leading to intracellular ROS and DNA damage then followed by caspase-3 activation and the releases of AIF and Endo G for causing apoptosis. We further showed that these events lead to cell death *via* caspase-dependent or -independent mitochondrial apoptosis with DNA fragmentation. Taken together, these data indicate that emodin might induce cell death in part through a caspase-dependent as well as in part through a caspase-independent (mitochondria-dependent led to AIF and Endo G and ER stress) apoptotic pathway by accumulation of ROS as a result of the disruption of the mitochondria in WEHI-3 cells as shown in Figure 6.

Emodin induced apoptosis in human promyeloleukemic HL-60 cells accompanied by activation of caspase cascade but independent ROS production [9]. However, other reports also showed that emodin induced apoptosis in human lung adenocarcinoma cells through a ROS-dependent mitochondrial signaling pathway [11]. Our previous studies also showed that emodin induced apoptosis in SCC-4 cells through the production of ROS and mitochondria-dependent pathways [15]. Currently, we showed that emodin induced apoptosis in murine leukemia WEHI-3 cells through ROS production, caspase-3 and mitochondria-dependent pathways. Therefore, it is raising the possibility that emodin may have some chemotherapeutic chance for human leukemia. However, the effects of emodin on leukemia *in vivo* provided no clear information.

Other reports [47] and our previous studies [48, 49] also showed the in vivo model through the mice intraperitoneally injected with WEHI-3 cells is well established. This mice model has been demonstrated as an ideal system for the study of potential therapeutic drugs (ATRA, aclacinomycin A, IL-6, G-CSF, and vitamin D3) which could induce *in vitro* differentiation of WEHI-3 cells in monocytic and granulocytic lineages [50–52]. Therefore, the purpose of this study was to examine the effects of emodin on WEHI-3 cells in BALB/c mice *in vivo*. Herein, our results showed that emodin inhibited spleen leukemia tumor growth in a WEHI-3 leukemia murine model. We observed that the size of the spleens decreased the emodin-treated leukemia groups. These observations were also seen in liver tissues, and there was a significant difference between the control and emodin-treated groups (Figure 7). Emodin also promoted the phagocytosis by monocytes and macrophages in PBMC and peritoneal cavity of WEHI-3 leukemia mice* in vivo *(Figure 9). 

In summary, emodin provokes apoptosis in mice leukemia WEHI-3 cells in vitro and tends to inhibit leukemia mice through stimulating phagocytosis *in vivo*. Our study is the first report and the in vitro the linkages of apoptotic cell death and the in vivo phagocytic activity of macrophages or monocytes were indeed needed for further investigation.
